# A putative *merR* family transcription factor Slr0701 regulates mercury inducible expression of MerA in the cyanobacterium *Synechocystis* sp. PCC6803

**DOI:** 10.1002/mbo3.838

**Published:** 2019-05-16

**Authors:** Deepak Kumar Singh, Bantu Lingaswamy, Tejaswi Naidu Koduru, Prakash Prabhu Nagu, Prakash Syama Sundar Jogadhenu

**Affiliations:** ^1^ Department of Biotechnology & Bioinformatics, School of Life Sciences University of Hyderabad Hyderabad India

**Keywords:** cyanobacteria, *merA* gene regulation, mercury tolerance, MerR transcription factor, *Synechocystis*

## Abstract

In cyanobacteria, genes conferring mercury resistance are not organized as *mer*‐operon, unlike in other bacterial phyla. *Synechocystis* contains only a putative MerR regulator, Slr0701, and a mercury reductase, MerA, located aside from each other in the genome. The *slr0701‐*mutant showed reduction in photosynthetic activity and reduced tolerance to mercury compared to the wild‐type. The incubation of wild‐type cells with HgCl_2_ resulted in the upregulation of *slr0701* and *slr1849* genes whereas mercury‐induced expression was not observed in the *slr0701‐*mutant. Slr0701 binds to a conserved *cis‐*regulatory element located in the upstream of *slr1849 *and *slr0701 *ORFs*. *The same element was also identified in the upstream of other cyanobacterial homologs. Slr0701 binds to *cis*‐regulatory element with faster association and slower dissociation rates in the presence of HgCl_2_. Although these genes were constitutively expressed, the addition of HgCl_2 _enhanced their promoter activity suggesting that mercury‐bound Slr0701 triggers induced expression of these genes. The enhanced promoter activity could be attributed to the observed secondary structural changes in Slr0701 in the presence of HgCl_2_. For the first time, we demonstrated the mechanism of *merA *regulation in a cyanobacterium, *Synechocystis*. Although *merA* and *merR* genes are distantly located on the cyanobacterial genome and distinct from other bacterial *mer*‐operons, the transcriptional regulatory mechanism is conserved.

## 1 INTRODUCTION

Certain metals, for example, silver (Ag), mercury (Hg), and tellurium (Te) are highly toxic to the living organisms and have microcidal property even at low concentration. Prokaryotes, fungi, and higher plants have evolved with molecular mechanisms to tolerate such harmful metal ions (Oves, Saghir Khan, Huda Qari, Nadeen Felemban, & Almeelbi, 2016). Investigations have demonstrated that mercury is readily accumulated by higher plants and causes severe damage to the cellular metabolism and physiology of the plants (Cargnelutti et al., 2006). Mercury hinders the photosynthetic electron transport and is exceedingly harmful as a result of the capacity of Hg^2+^ to bind with proteins (Rai, Agrawal, & Agrawal, 2016). Once, in the cell, Hg^2+^ forms covalent bonds with cysteine residues of proteins, it drains antioxidants. These mercuric ions in the aquatic environment persist for very long period in the sediments (Randall & Chattopadhyay, 2013). However, the microorganisms evolved with a molecular mechanism to resist the inorganic and organic forms of mercurial compounds involving *mer*‐operon.

Mercury‐tolerant bacterial strains reported till to date contain *mer*‐operon in their genomes, which code for proteins involved in mercury detoxification. The main open reading frames, *merR*, *merA*, and *merP*, code for a transcriptional regulator, mercuric reductase, and a periplasmic mercury binding protein, respectively. In some bacterial species, additional genes such as *merB* code for organo‐mercurial lyase, *merD* an additional transcriptional regulator, *merE* and *merF*, auxiliary transporters are present. The enzymatic reduction of the mercuric ions to elemental mercury, catalyzed by mercury reductase, is the main detoxification mechanism in bacteria (Barkay, Miller, & Summers, 2003). This key detoxification protein reduces the toxic Hg (II) to Hg (0). As a result of its low dissolvability in water and moderately high‐vapour weight, the elemental mercury is discharged from the cell (Barkay et al., 2003; Silver, 1996). The biochemical basis of protection from inorganic‐mercury compounds, for example, HgCl_2_ seems to be similar in few bacterial species. Studies have shown that a wide range of microorganisms such as *Shigella flexneri*, *Pseudomonas aeruginosa*, *Serratia marcescens*, *Xanthomonas *sp., and *Staphylococcus aureus *possess *mer*‐operon. However, when *mer* genes were compared, the number and their operonic organization differ among various bacterial genera (Barkay et al., 2003; Hobman, Wilson, & Brown, 2000).

Cyanobacteria, which occur in almost all environmental niches contain genes that code for a putative MerR‐like transcriptional regulator and MerA, mercury reductase. In *Synechocystis* sp. PCC6803 (hereafter referred as *Synechocystis*), two putative MerR family transcription factors are predicted. One of them, Slr0794 has been reported to be involved in the regulation of genes related to Ni^2+ ^toxicity (García‐Domínguez, Lopez‐Maury, Florencio, & Reyes, 2000). Several MerR transcription factors, though they are called as mercury‐resistant regulators and classified under MerR family, are also known to be involved in resistance to toxic metals other than mercury. For example, ZntR (Brocklehurst et al., 1999) in *Escherichia coli* and PbrR in *Ralstonia metallidurans* CH34 (Harley & Reynolds, 1987) are MerR family members involved zinc and lead resistance, respectively. In *Synechocystis*, Slr1849, codes for MerA and biochemically it was demonstrated to be a mercury reductase (Marteyn et al., 2013). However, the *mer*‐operon organization and regulation in cyanobacteria are poorly understood. Being photosynthetic bacteria, progenitors of higher plant chloroplasts and occurring in almost all habitats on the earth, it is important to unravel the regulatory mechanism of mercury tolerance in cyanobacteria. In this study, we show that the *mer* genes of cyanobacteria are phylogenetically distinct from other bacterial phyla. We also demonstrate a mercury‐induced regulation of *merA* gene by a putative MerR family protein in *Synechocystis*.

## 2 MATERIALS AND METHODS

### 2.1 Bacterial strains and culture conditions

*Synechocystis*, a glucose‐tolerant strain that was initially acquired from Dr. J.G.K. Williams (Dupont de Nemours, Wilmington, DE, U.S.A.), served as the wild‐type. Wild‐type cells were cultured photoautotrophically at 34°C in BG‐11 added with 20 mM HEPES/NaOH, pH 7.5 under consistent light at 70 μE m^−2^ s^−1^ of photons as presented earlier (Krishna et al., 2013). The *slr0701‐*mutant cells in which the *slr0701* ORF was inactivated by inserting a spectinomycin (sp^r^) cassette was also cultured similarly as described above with an exception that the BG‐11 medium contained spectinomycin at 25 μg/ml in pre‐culture. The growth of the cells was monitored by estimating the absorbance at 730 nm in a spectrophotometer (NanoDrop™, 2000C, Thermo Fisher Scientific).

### 2.2 Identification and phylogenetic analysis of putative mercury responsive genes

Protein sequences involved in mercury resistance from *Bacillus megaterium* and *E*. *coli* were used as queries for searching homologs in *Synechocystis *genome, which is publicly available at “cyanobase” http://genome.microbedb.jp/cyanobase/ (Kaneko et al., 1996). Slr0701 and Slr1849 protein sequences from *Synechocystis *were used to search for homologues from other cyanobacteria. The homolog protein sequences were obtained from the KEGG and NCBI databases (http://www.genome.jp/dbget-bin/ and http://www.ncbi.nlm.nih.gov/gquery/) for building phylogenetic tree. Phylogenetic connections were deduced by phylogeny examination using http://www.phylogeny.fr/advanced.cgi (Dereeper et al., 2008).

### 2.3 Generation of *slr0701*‐mutant and *slr0701* ^+^ complement strains

We have generated a *slr0701‐*mutant of *Synechocystis* by inserting a Ω‐spectinomycin resistant (sp^r^) cassette within the *slr0701 *ORF. A DNA fragment containing the *slr0701* ORF with 602 bp upstream and 325 bp downstream flanking regions were amplified by PCR using sequence‐specific primers: *slr0701*‐FP (5′‐CAC CCT GGT TTG ATC AAT ACT CC‐3′) and *slr0701*‐RP (5′‐CGA TCG CCC ATC TGT GTT GAA G‐3′). The PCR amplified fragment (1340bp) was ligated to a linear T‐vector (InsTAclone™ PCR Cloning Kit, #K1214). The resultant plasmid pTslr0701 was used to inactivate the *slr0701* gene by performing restriction digestion at *Hpa*I site. The sp^r^ gene cassette was PCR amplified with specific primers *Sp*‐F (5′ AAACTTTTTAAATCCTTAATTATTTGCCCACTAAAC 3′), *Sp*‐R (5′ ATCAAAGTT TAA AACTCC CCC AGG GTC TTA GTT C 3′) using *ΔcrhR *genomic DNA in which sp^r ^cassette was previously used to inactivate *crhR *gene (Prakash et al., 2010). The *Dra*I site in the Sp^r^ specific primers was underlined. The *Dra*I‐digested Sp^r ^cassette was cloned at the *Hpa*I‐digested pTslr0701 DNA construct by blunt end ligation. The final plasmid, pTslr0701*::*sp^r^ in which the *slr0701 *ORF was disrupted by Sp^r ^cassette was used to transform wild‐type *Synechocystis* cells. The site of insertion of the Sp^r^ cassette was confirmed by sequencing the pTslr0701*::*sp^r^ DNA construct using the *slr0701*‐F primer. The segregation analysis was performed by PCR amplification to check the extent of replacement of wild‐type copies of *slr0701* with the pslr0701*::*sp^r ^in *slr0701*—mutant strain was verified by PCR amplification using genomic DNA as a template.

The *slr0701^+^*complement strain was generated to check whether the phenotype change observed in *slr0701 *mutant was due to inactivation of *slr0701 *gene. The PCR‐amplified *slr0701* ORF with flanking regions as mentioned above was used for making complement. The resulting 1,340 bp DNA fragment was cloned by blunt end ligation at *Sma*I site located within the kanamycin (kan^r^) cassette into a cyanobacterial replicative vector pVZ321vector (Zinchenko, Piven, Melnik, & Shestakov, 1999). The recombinant vector, pVZ*‐*slr0701, was introduced into *slr0701‐*mutant by triparental mating as described in Zinchenko et al., 1999. We transferred the recombinant replicative pvz*‐*slr0701 into the recipient *slr0701‐*mutant strain by conjugal mating with donor and helper *E. coli *strains. *E. coli‐DH5 *α carrying pvz‐slr0701 was served as a donor strain. The recipient, donor, and helper strains were mixed in a ratio of 10:1:1, on a membrane filter (Millipore, Catalog No: GSWP 04700), and incubated overnight on BG‐11 solid agar supplemented with 5% LB under dim light for conjugal transfer. The colonies thus developed were selected with antibiotic selection pressure of 35 µg/ml of chloramphenicol (Cm^r^) on BG‐11 agar medium in addition to 25 µg/ml spectinomycin. The strain thus generated was named *slr0701* ^+^.

### 2.4 Growth and viability of *Synechocystis *strains under HgCl_2_

The phenotypic characterization of wild‐type and the *slr0701 *mutant strains was performed in the presence of different concentration of iron, copper, zinc, cobalt, and mercury. About 50 ml of the wild‐type, *slr0701*‐mutant and *slr0701^+^*strains were grown till mid‐log phase, that is, OD_730nm_ equals to 0.6 and were collected by centrifugation at 3,500 rpm for 5 min using swing‐bucket rotor (Eppendorf, R5804). The cell pellets were washed twice with liquid BG‐11 medium and resuspended in a small volume of fresh BG‐11 medium to adjust the final density of the resuspended cells equivalent to 10 at OD_730nm_. The cell suspensions were serially diluted five times, taking each time 500 µl of resuspended cells into 500 µl BG‐11 medium (in 1:1 ratio). Finally, 50 µl of diluted cell suspensions was spotted onto solid BG‐11 agar plate containing different concentrations of trace elements as mentioned above. Similarly, 50 µl of diluted cell suspensions was spotted onto solid BG‐11 agar plate containing different concentrations of HgCl_2_ (0–500 nM). To check the effect of mercury on their growth and viability, growth was monitored for 5 days from the day of spotting on the BG‐11 agar plates and photographed on 5th day.

### 2.5 Measurement of photosynthetic electron transport rate

Photosynthetic oxygen evolution was estimated in 1 ml of cell suspension (OD_730_ = ~1, around 5 μg/mL chlorophyll), utilizing an oxygen electrode (Oxygraph plus, Hansatech Instruments Ltd., Norfolk, England). The cells were grown photoautotrophically under continuous air bubbling by illuminating continuously with 70 μE m^−2^ s^−1^ light. PSII activity was measured in presence of 1.0 mM p‐benzoquinone (PBQ) as described earlier (Sireesha et al., 2012). Photosynthetic oxygen evolution was recorded at 1,000 μE m^−2^ s^−1^ light. Three independent cultures of wild‐type and *slr0701*‐mutant cells were estimated with or without HgCl_2_. Cultures were grown till 0.6 OD at 730 nm and measured the oxygen evolution and the data was used as a reference. Then 500 nM of HgCl_2 _was added to a final concentration of the cultures and measured oxygen evolution at 12 hr and 24 hr.

### 2.6 Measurements of chlorophyll *a* fluorescence

We recorded chlorophyll *a* fluorescence of *Synechocystis *cell suspensions with a continuous excitation PEA fluorometer (PEA, Hansatech, King's Lynn, Norfolk, UK). The PEA fluorometer provides continuous excitation at 650 nm (3,000 μE m^−2^ s^−1^; Δλ = 22 nm). It perceives fluorescence at wavelengths above 700 nm (50% transmission at 720 nm) and records it continuously from 10 μs to 300 μs. The fluorescence curves were recorded using wild‐type and *slr0701*‐mutant cells as described (Sireesha et al., 2012). Cultures were grown till OD at 730 nm reached to 0.6, and then measured the chlorophyll *a* fluorescence. These data were used as a reference. Then 500 nM of HgCl_2 _was added to a final concentration of the cultures and measured fluorescence at 12 hr and 24 hr.

### 2.7 Transcript analysis of mercury responsive genes

Wild‐type and *slr0701*‐mutant cells were cultivated at 70 μE m^−2^ s^−1^ of light. 50 ml of the cells were killed immediately by the addition of 50 ml equal volume of cold 5% w/v, phenol in ethanol, and then total RNA was isolated as described earlier (Srikumar et al., 2017). The RNA was treated with DNase I (Cat. No. 89836, Thermo Fischer Scientific) to remove the DNA contaminants. One‐microgram of RNA from total RNA was converted to cDNA using Takara Kit (Cat. no. 6110A). RNA was isolated from wild‐type and *slr0701*‐mutant cells before and after treatment with HgCl_2_. This RNA was utilized for cDNA preparation with the Prime Script™ first strand cDNA Synthesis Kit (Cat. no. 6110A). qRT‐PCR was done using the SYBR^®^ Premix Ex Taq™ II (TliRNase H Plus; feline. no. RR820A). Every reaction was completed in a 25 μl volume containing 12.5 μl of Power SYBR Green Master Mix, 0.2 μM of specific primers.

(Table 1) and 5 μl of diluted cDNA was kept in triplicates for running qRT‐PCR (Mx3005P, Agilent Technologies). The instrument was conditioned for 95°C for 10 min, and then 40 cycles of 30 s at 95°C, 60 s at 60°C, and 60 s at 72°C. For every reaction, the melting curves were investigated and the PCR product was analyzed on an agarose gel with the end goal to affirm the specificity of the RT‐PCR. Expression levels were normalized using the *gap1* gene as an internal reference.

**Table 1:** Primers used for qRT‐PCR

Gene	Forward primer (5′‐3′)	Reverse primer (5′‐3′)
*slr0701*	GTTTCATTCGTCATGCCAAGG	CTTTGATTCCACTTTCGAGCTG
*slr1849*	TGATTTTCCCGCTGTGATGG	TCCCGAAATCTTTGAGCAGAG
*slr0884 (gap1)*	TCGCCTACCGTTGGAGCC	GGGCACAATGGCTTCAACAA

### 2.8 Overexpression and purification of Slr0701

PCR amplification of *slr0701* ORF was performed, with the specific primers slr0701‐ExF (5′‐gtacGC TAG CGT GAG CAT TAT GTT AAC CGT CAG C‐3′) and slr0701‐ExR (5′‐gcatGAA TTC CTA AGT CAA CTG CTC ATT TAA CAA AC‐3′). The *Nhe*I and *Eco*RI restriction enzyme sites are underlined. The PCR product was purified and then cloned into pET‐28a (+) at the *Nhe*I and *Eco*RI sites to produce pET‐slr0701 by site‐specific restriction cloning. The C‐terminally 6x‐His‐labeled Slr0701 protein was overexpressed in Rosetta™ (DE3), which had been transformed with pET‐slr0701 and was induced with 0.4 mM final concentration of isopropyl β‐D‐1‐thiogalactopyranoside (IPTG). The *E. coli* cells were pelleted with centrifugation at 4,000 rpm for 5 min at 4°C. The cell‐pellet was resuspended in the lysis solution containing 6 M urea, 50 mM Tris, 300 mM NaCl, 10 mM Imidazole/pH 8, sonicated for 15 min at 30% amplitude, 30 cycles at a hold of 30 s. each. Homogenized cells were centrifuged at 14,000 rpm for 30 min at 4°C to separate soluble and insoluble fraction of lysed cells. The soluble fraction collected was loaded onto HIS‐Select TM Nickel‐Affinity gel, 1 ml (Cat. No. P6611, Sigma‐Aldrich) and after that washed with 2‐bed volume wash solutions containing 6 M urea, 50 mM Tris, 300 mM NaCl, 20 mM Imidazole/pH 8 to washout impurities, and undesirable protein. 6x‐His‐Slr0701 protein was eluted in an elution solution containing 6 M urea, 50 mM Tris, 300 mM NaCl, and 200 mM Imidazole/pH 8. These elutes were analyzed on 12% SDS‐PAGE. The Slr0701 protein was pooled in elution solution of pH 8 and serially dialysed at 4°C for seven times using the initial solution containing 50 mM Tris, 300 mM NaCl, and 100 mM Imidazole. At each round of buffer replacement, urea and imidazole concentrations were gradually decreased to attain the final buffer concentration of 50 mM Tris and 300 mM NaCl.

### 2.9 Western blotting analysis

Western blotting experiment was performed as presented earlier with some modifications (Prakash et al., 2010). Polyclonal antibodies induced in rabbit against 6x‐His–Slr0701 protein was utilized as primary antibody, and an HRP‐linked antibody induced in goat against rabbit IgG was utilized as the secondary antibody. Wild‐type cells were grown with and without 500 nM HgCl_2, _and the cells were harvested for protein extraction at different time intervals. Protein samples were separated using SDS‐PAGE and were blotted onto PVDF membrane (Cat. No. IPVH00010, Immobilon‐P; Merck Millipore) with a semi‐dry transfer apparatus (TE77‐PWR semi‐dry transfer unit, GE Healthcare). The levels of Slr0701 were detected immunologically with the ECL Plus, immunoblotting system (Cat. No. 1705060‐61, Bio‐Rad). We used anti‐rabbit secondary antibodies conjugated to horseradish peroxidase (1:5,000) for detection. Blot was scanned using Bio‐Rad chemidoc (XRS^+^) and analyzed with Image Lab software (Bio‐Rad).

### 2.10 Prediction of *cis‐*regulatory elements using MEME suite

Intergenic DNA regions of cyanobacterial orthologs obtained from blastP were taken and submitted to MEME (Motif Extraction by Multiple Expectation Maximizations) version 4.3.0. MEME was run using the default parameters to find maximum of three motifs per sequence with the distribution of zero or one occurrence per sequence (Bailey & Elkan, 1994).

### 2.11 Gel retardation assays

The *slr0701* and *slr1849* upstream DNA regions were PCR amplified using oligonucleotide primers *pslr0701‐FP‐B*: 5′CCC TCC CAT TTC TTC TCT GTC TCT ACT AC3′ and *pslr0701‐RP‐B*: 5′GGG GTT AGG TAG GCG ATC GCC AAA TTA C3′, and *pslr1849‐FP*–B: 5′CGT GAA TGT AAA TGA AAA AAC AAG CG3′ and *pslr1849‐RP‐B: *5′ACT TAG AAT TGC TGA TTG CCG TAT TAC 3′. The sizes of amplified PCR products corresponding to *slr0701 *and *slr1849 *upstreams were 231 bp and 230 bp, respectively. The binding reactions were performed in the 20 μl reaction volume containing 80 ng of upstream DNA and 4 µl of the binding buffer with different concentration of 6x‐His‐Slr0701. The mixtures were incubated for 30 min with and without 50 µM HgCl_2_ at 25°C. The reaction mixtures were loaded on a non‐denaturing 6% polyacrylamide gel. Electrophoresis was carried out at 4°C and 200 V in 0.5X Tris‐borate/EDTA. Gels were washed and incubated in a clean plastic container with sufficient 1X SYBR^®^ Green EMSA staining solution about 50 ml with continuous, gentle agitation at 50 rpm for ~20 min, protected from light. For visualization, gel was washed twice with 150 ml of ddH_2_O for ~10 s to remove excess stain. The gel was scanned using Bio‐Rad chemidoc (XRS^+^) and analyzed with Image Lab software (Bio‐Rad).

### 2.12 Biomolecular interaction analysis by SPR

All the experiments in surface plasmon resonance (SPR) analysis were performed at 25°C using Biacore T200 instrument (GE Healthcare Life Sciences). To study the interactions under real‐time conditions between 6x‐His‐Slr0701 protein and P*_slr0701_*, a DNA fragment covering the predicted conserved DNA binding motif by MEME, was synthesized (Integrated Device Technology, Inc.). Biotinylated oligonucleotide containing a sequence 5′Biosg/TAC TAC AAT ATA TTC CCT ATA CTT AGG TAT AAG GTT GTA GGT TGA TAT AG3′ and the complementary sequence 5′CTA TAT CAA CCT ACA ACC TTA TAC CTA AGT ATA GGG AAT ATA TTG TAG TA3′ were hybridized to obtain double‐stranded DNA duplex, P*_slr0701_*. SA sensor chip (GE Healthcare Bio‐Sciences AB, Uppsala, Sweden) was kept at room temperature for atleast 30 min before use. The instrument was first primed three times with HBS_EP+ running buffer. The flow cell 3 (FC 3) was used as the reference cell (without biotinylated ligand) and flow cell 4 (FC 4) was used for the immobilization. According to the manufacturer's instructions before immobilization the SA chip was conditioned for three consecutive injections of 1 minute duration each with 1 M NaCl in 50 mM NaOH (activation buffer), followed by immobilization of the DNA. Reference cells were washed with 50% isopropanol in 1 M NaCl and 50 mM NaOH. After surface activation of the sensor chip, immobilization of 500 nM of biotinylated *P_slr0701 _*dissolved in a final volume of 128 µl HBS_EP+ buffer was performed.

Slr0701 protein was chosen as an analyte to screen the protein–DNA interactions in HBS_EP+ running buffer. The method was standardized with varying concentration of Slr0701 from 2 to 32 µM with and without 62 µM of mercury chloride. The experiment was performed in triplicates. At the end of each dissociation period, the sensor chip was regenerated using 10 mM Glycine‐HCl pH 2.0 for a contact time of 30 s. with a flow rate of 30 µl/min followed by a subsequent wash with running buffer for each cycle, passing through FC3 and FC4 in series, recording the reference subtracted signal. Biacore T200 evaluation software version 2.0 was used for data analysis, 1:1 Langmuir model fit was used for determining the binding kinetics.

### 2.13 Analysis of P*_slr0701 _*and P*_slr1849 _*promoter activity

To monitor the activity of promoters, we fused LuxAB, reporter gene coding for luciferase to the promoter of *slr0701* and *slr1849.* The modified pSyn2030‐2031P_Cot_ vector was used to construct the desired promoter clones within *slr2030*‐*slr2031* neutral site. The vector was linearized using inverse PCR technique with specific primers FP: 5′ AGA AGG AGC GTC AGA TCT CAT ATG C3′, RP: 5′ GAG ACG TTG ATC GGC ACG TAA G3′ and the upstream DNA fragments were PCR amplified with the infusion primers specific to *pslr0701*FP: 5′ GAT CAA CGT CTC ATT ACA GTC GAG AAC TAA GAC AA3′, *pslr0701*RP: 5′ GAT CAA CGT CTC ATT ACA GTC GAG AAC TAA GAC AA3′ and *pslr1849* FP: 5′ GAT CAA CGT CTC ATT ACT TAG AAT TGC TGA TTG CC3′, *pslr1849*RP: 5′ ATT TCC AAA CTT CAT TTA AGG ATT AAT TGT TTA ATG3′ 100 μl wild‐type and *slr0701‐*mutant *Synechocystis *cells were transformed with 1 μg of plasmid DNA containing either P*_slr0701 _*or P*_slr1849_*. The cells were incubated for 8 hr in the dim light. Subsequently, cells were plated on BG‐11 chloramphenicol (Cm) gradient plate. After 10 days, the colonies from the gradient plate were picked and plated onto to BG‐11 agar plate containing chloramphenicol at a concentration of 10 µg/ml. For complete segregation, Cm‐resistant colonies were grown at increasing Cm concentrations (up to 50 μg/mL) and finally transferred into the liquid BG‐11 medium. In case of *slr0701‐*mutant cells, screening was performed in the presence of spectinomycin. Luminescence of the luciferase reactions was induced by the addition of 500 nM HgCl_2_ to the cyanobacterial cell suspension. Light emission was monitored in a photomultiplier based luminometer (BioOrbit, Labsystems) following the addition of N—decanal (50 mM decanal in methanol/water (50%, v/v), the final concentration in the suspension was 1 mM). We used the *LuxAB *plasmid without P*_slr0701 _*and P*_slr1849_* in the wild‐type and *slr070—*mutant as a control to evaluate the metal‐induced regulation of *slr0701* and *slr1849* promoters.

### 2.14 CD spectroscopy

The upstream of *slr0701* was amplified by PCR using specific primers FP 5′CCC TCC CAT TTC TTC TCT GTC TCT ACT AC3′ and RP 5′GGG GTT AGG TAG GCG ATC GCC AAA TTA C3′ from the genomic DNA of *Synechocystis*. A 231 bp PCR amplified product was purified and used for CD spectral analysis. CD spectra were recorded with a Jasco J‐1500 spectropolarimeter. Far UV‐CD spectra of 20 µM of Slr0701 were recorded in the presence of varying concentrations of HgCl_2 _ranging from 20–200 µM. The ellipticity value at 222 nm was plotted against the concentration of HgCl_2_. Further, the near UV‐CD spectra of Slr0701 protein was analyzed in the presence of DNA, without and with 25 µM of mercuric chloride. The protein and DNA concentrations used were 5 µM each. The spectra were collected with a scan speed of 20 nm/min and an average of three scans was calculated.

## 3 RESULTS

### 3.1 Diversity in the genetic organization of *mer *genes in cyanobacteria

**Figure A1:**
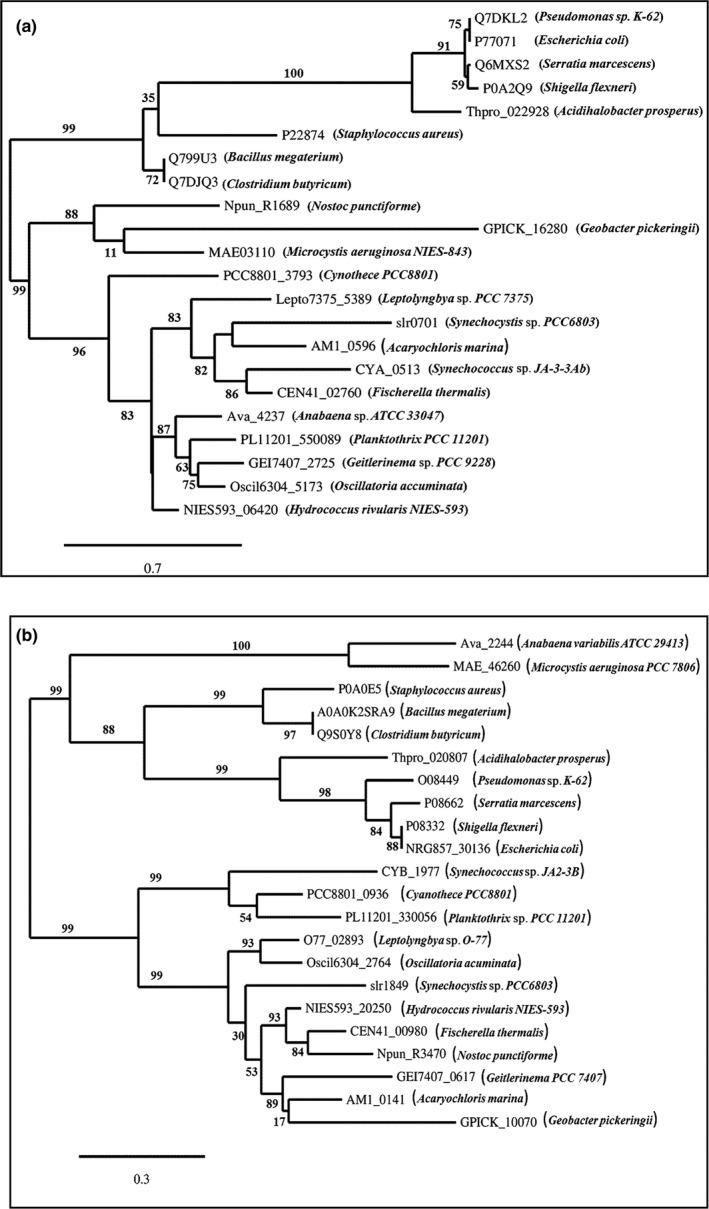
Phylogenetic relationship of Slr0701 and Slr1849 with their closely related orthologs. The complete amino acids sequences of Slr0701 and Slr1849 and their orthologs were taken, phylogenetic relationship was deduced. All investigated cyanobacterial and bacterial strains are presented in the figure. The well‐studied *Bacillus megaterium*, MerR and MerA with five other proteins from different organisms more closely related to these Mer proteins were also included. The percentage of replicate trees in which the associated taxa clustered together in the bootstrap test (500 replicates) is shown next to the branches. The tree is drawn to scale, with branch lengths in the same units as those of the evolutionary distances used to infer the phylogenetic tree. The evolutionary distances are given in the number of amino acid substitutions per site as indicated by the scale bar representing 0.7 for *slr0701 *(a) and 0.3 for *slr1849 *(b) substitutions per amino acid

To identify the mercury responsive genes in *Synechocystis, *sequences of well‐characterized *mer* proteins from certain gram‐positive and gram‐negative bacteria were used as queries and blastP search was performed against *Synechocystis *genome database as described in the experimental methods. The best hits of blastP search using well‐characterized MerR and MerA were a putative *merR* transcriptional regulator and a mercury reductase encoded by ORF numbers *slr0701 *and *slr1849*, respectively. The evolutionary relationship of Slr0701 and Slr1849 with their respective orthologs is shown in Appendix information Figure A1. The phylogenetic trees demonstrate their presence across different bacterial and cyanobacterial genera. The homologs for Slr0701 and Slr1849 proteins were identified mainly in freshwater cyanobacterial species and some other non‐photosynthetic bacteria isolated from soil as well. Though several of these cyanobacterial species were originally isolated from freshwater ponds, they were observed to exist in soil crusts also (Tirkey & Adhikary, 2005). It was also observed that both, MerR and MerA homologs from cyanobacterial species formed a separate clade (Figure A1)*. *Homologs for Slr0701 and Slr1849 proteins were also present in *Geobacter pickeringii*, a metal‐reducing isolate from sedimentary kaolin deposits (Shelobolina et al., 2007), and *Acidihalobacter prosperus*, isolated from geothermally heated sea sediments (Khaleque, 2017). The orthologs from *G. pickeringii *formed a separate clade along with the cyanobacterial *mer *genes. Notably, Slr0701 and Slr1849 of *Synechocystis *showed 53.2% and 46% sequence similarity to a well‐studied *B. megaterium *MerR and MerA homologs, respectively (Wang et al., 1989). Interestingly, Slr1849 showed 70.2% and 43.1% sequence similarity with the MerA of metal‐reducing bacterial species, *G. pickeringii *and *A. prosperus*, respectively. But, the putative regulator, Slr0701, showed only 37.3% and 44.8% similarity to MerR of these metal‐reducing bacterial species. This suggests that the cyanobacterial MerA might have horizontally transferred and independently acquired from metal reducing *Geobacter *species*.*

**Figure A2:**
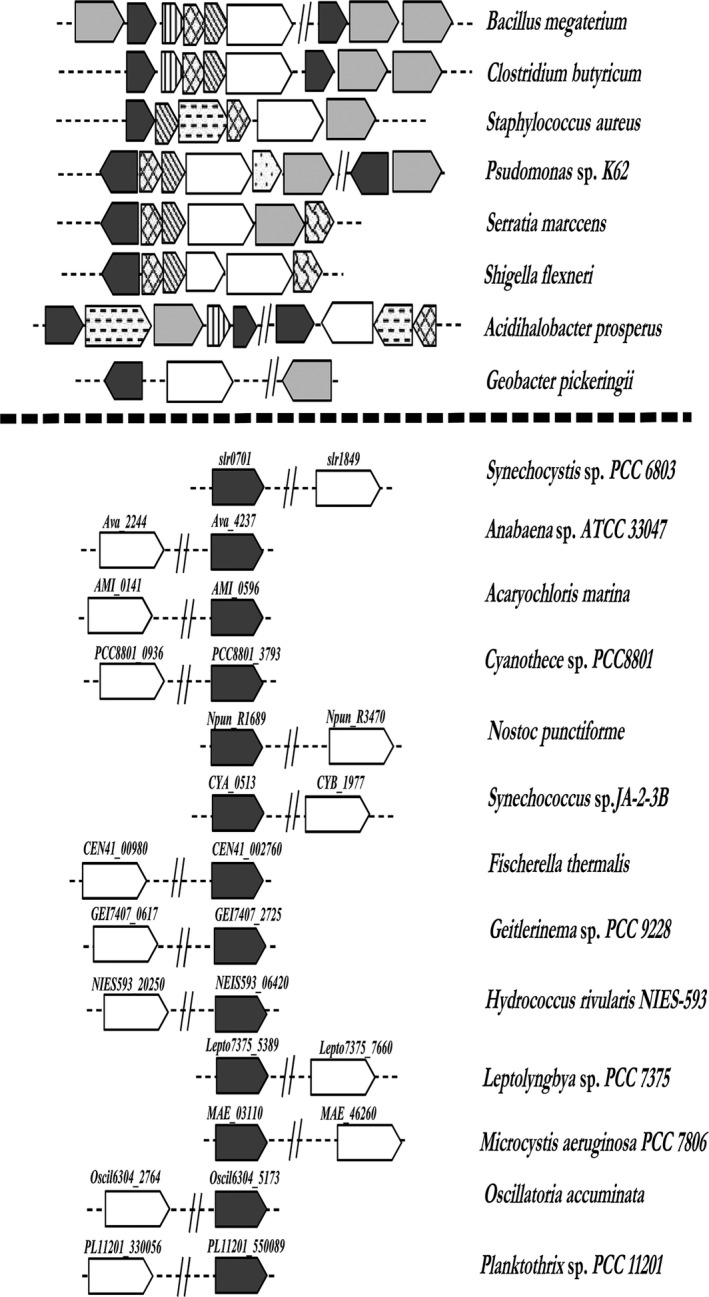
Comparison of *mer‐*operon organization in different bacterial and cyanobacterial species. *merR *(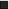), Transcprional regulator*; merG *(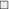)*, merT*(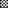),* merP*(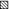),* merC*(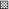),* merE*(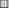)*, *periplasmic proteins; *merA*(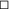)*, *mercury reductase; *merB* (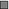), organo‐mercurial lyase and *merD *(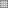) alternative transcriptional regulator. The reading frames are shown as arrows to indicate ORFs. The *mer *genes shown below the bold dash line belong to cyanobacterial species. (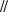) indicates that the genes are located distantly from each other in the genome

In bacteria, proteins involved in resistance to mercury are encoded by mercury responsive transcription factor; *merR, *an inorganic‐mercury reductase; *merA,* alternative transcription factor; *merD, *periplasmic transporters; *merP, merT *and *merC, *and organo‐mercurial lyase; *merB*. These genes are organized as *mer* operon in most of the bacterial species reported to date (Barkay et al., 2003). Comparison of the organization of *mer* genes from a diverse range of bacterial species has revealed considerable similarity in their genetic organization (Figure A2). Almost all these operons contain a regulatory gene, *merR*. In gram‐positive bacteria, the *mer* genes including *merR *form a single operon. Downstream to the regulator, *mer*R other *mer* structural genes *merA*, *merD, merP,* and *merT *are located in gram‐positive bacteria. In some gram‐positive bacteria, an additional ORF, *merC,* is also part of the operon. In some gram‐negative bacteria, *merR *is divergently located to other *mer* structural genes which form an operon without *merR*. In certain bacterial species, such as *B. megaterium *and *Pseudomonas* sp. K62, an organo‐mercurial lyase, *merB *was also reported as a part of the *mer‐*operon (Kiyono, Omura, Inuzuka, Fujimori, & Pan‐Hou, 1997; Wang et al., 1989). In some bacterial genomes, more than one copy of the structural gene for *merA*, *merB* and *merR *were reported (Khaleque, 2017; Kiyono et al., 1997; Wanget al., 1989). In *Synechocystis*, only *merA *and *merR *genes exist and were distantly located in the genome and all other *mer* genes, such as *merC, merD, merP, merT, merG, merE, *and *merB *were not detected*. *It is important to note that not only in *Synechocystis, *but also in all other cyanobacterial species reported to date, *merR *and *merA *are distantly located in their genomes unlike in other bacterial species (Figure A2). However, the mechanism of gene regulation in response to mercury metal toxicity has not been reported in any cyanobacteria. Since homologs of the Slr0701 and Slr1849 appear to be well conserved among different aquatic cyanobacteria and relatively more similar to the metal‐reducing bacterial species*, *it appears likely that the Slr0701 might be a regulatory gene in metal stress response.

### 3.2 Complete targeted inactivation of a gene, *slr0701 *coding for putative MerR

**Figure 1:**
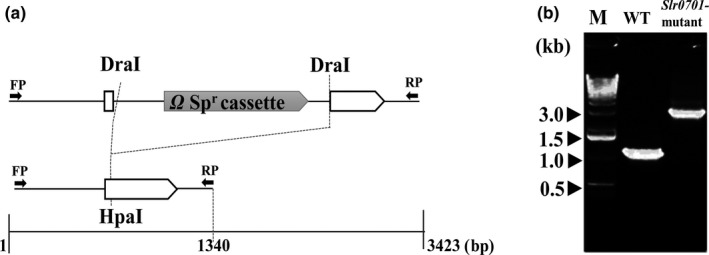
Strategy for disruption of the *slr0701 *gene in the genome of *Synechocystis*. (a) Schematic representation of the genotype of the *slr0701* mutant. A 1,340 bp DNA fragment covering *slr0701 *gene and a 2083 bp DNA fragment containing Sp^r^ gene are drawn to scale. The sp^r^ and *slr0701* ORFs are shown in the filled and open arrows, respectively. Small arrows indicate positions of PCR primers used to amplify the region and to verify gene replacement. FP: Forward primers; RP: Reverse primers. (b) PCR analysis of wild‐type and *slr0701‐*mutant cells with the primers indicated in (a). M represents 1‐kb DNA ladder (Invitrogen™ Life Technologies; Cat. No. 15615‐016)

To elucidate the regulatory role of Slr0701, the ORF was inactivated as shown in the schematic representation (Figure 1a)*. *The extent of replacement of wild‐type copies of the *slr0701 *with that of disrupted copies of *slr0701::*sp^r ^was confirmed by comparing the sizes of the amplified PCR products. When the genomic DNA from wild‐type was used as a template for PCR amplification with specific primers (*slr0701‐*F and *slr0701*‐R), a PCR product of 1,340 bp representing the *slr0701 *ORF was amplified including the upstream and downstream regions. In contrast, when the genomic DNA isolated from *slr0701‐*mutant were used as a template with the same set of primers, a 3,423 bp PCR product was amplified corresponding to the wild‐type fragment of 1,340 bp including the inserted Ω‐spectinomycin gene (sp^r^) cassette of 2083 bp (Figure 1b).

### 3.3 *slr0701‐*mutant is more sensitive to HgCl_2_ treatment compared to wild‐type

**Figure 2:**
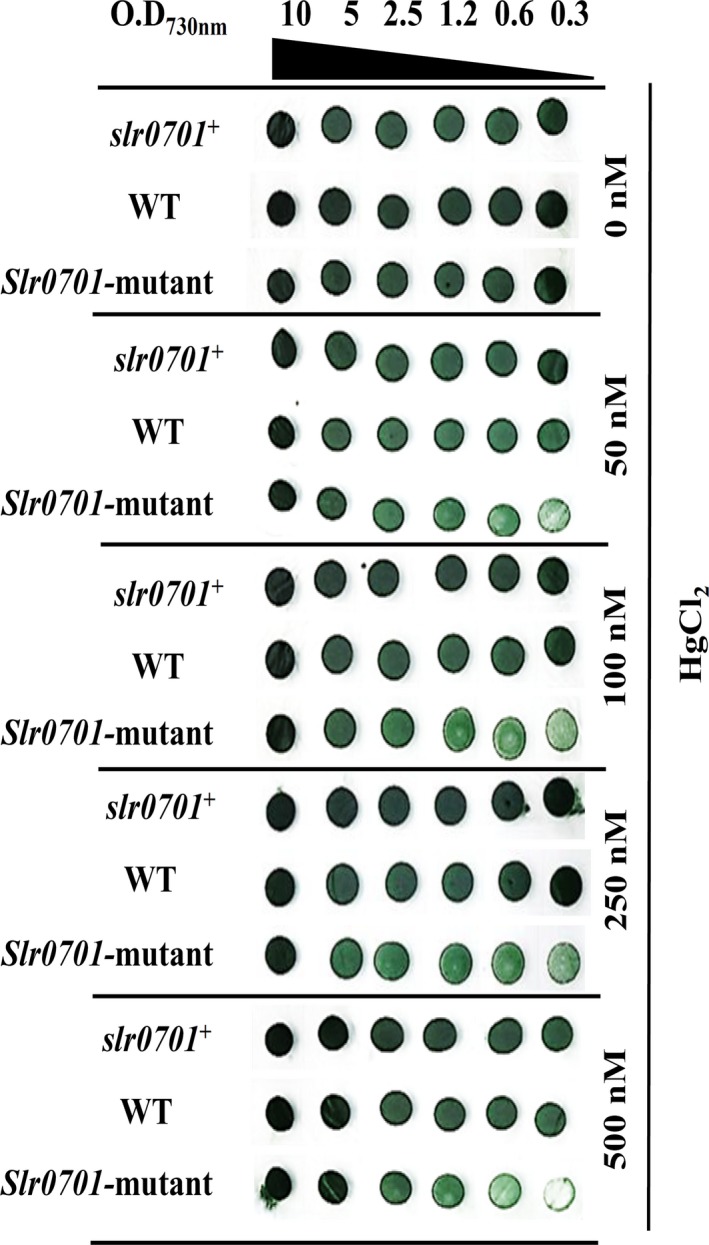
*slr0701 *is involved in mercury resistance. Tolerance of wild‐type, *slr0701‐*mutant and *slr0701^+^*strains to mercury was examined. As indicated serially diluted cultures were spotted onto solid BG‐11 agar plate and also on HgCl_2_ supplemented BG‐11 agar plates with the indicated concentrations. Plates were photographed after 5 days of incubation under continuous illumination of 70 μE m^−2^ s^−1^ of photons

**Figure A3:**
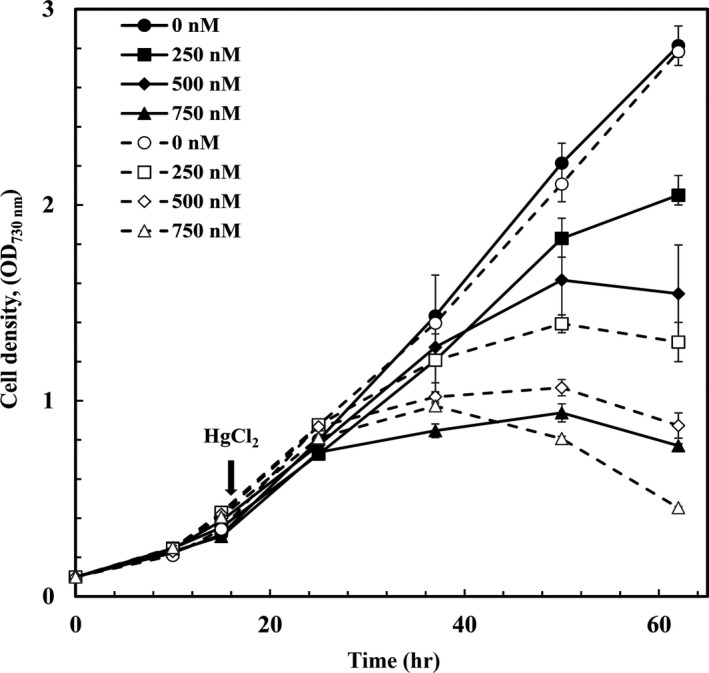
Effect of HgCl_2 _on growth of *Synechocystis *wild‐type and *slr0701‐*mutant strains. For monitoring the growth in the presence of HgCl_2,_ cultures were initially allowed to grow till 16 hr as described in methods, and then HgCl_2_ was added to a final concentration of 250 nm, 500 nm and 750 nm to the respective culture tubes. Optical density at 730 nm was measured at regular intervals. Filled symbols represent growth of wild‐type *Synechocystis* cultures and open symbols represent *slr0701*‐mutant cultures. The mean of three experimental values is presented with standard error

Since, *slr0701 *codes for a putative MerR type transcriptional regulator, we investigated the effect of trace metals on the viability of *slr0701*‐mutant cells in comparison with wild‐type. Initially, to find the involvement of Slr0701 in metal resistance, we tested the growth of wild‐type and *slr0701*‐mutant strain in which *slr0701 *gene was inactivated on a solid BG‐11 media containing various metals as described in experimental procedures. Both wild‐type and *slr0701‐*mutant cells revealed similar profiles of growth at different metal ions tested except the growth on the BG‐11 solid plate containing HgCl_2_ (data not shown). In order to confirm that the slow growth phenotype seen in *slr0701‐*mutant was due to HgCl_2_, we compared the growth profiles of wild‐type, *slr0701‐*mutant and *slr0701^+^‐*complemented cells in the absence and presence of HgCl_2 _(Figure 2). All the three strains exhibited similar growth profiles when serially diluted cultures were spotted on a BG‐11 agar plate without HgCl_2_. However, with increasing concentrations of HgCl_2_ in the BG‐11 solid agar plate, *slr0701* mutant exhibited a slow growth phenotype compared to wild‐type. At 500 nM of mercuric chloride, almost no growth of the mutant cells was observed in the spot area, at which 5‐times diluted cells were deposited (Figure 2). The complementation of *slr0701‐*mutant cells with a functional Slr0701 expressed from pVZ321‐slr0701 restored growth, and appeared similar to that of wild‐type growth in the presence of mercuric chloride. We also tested the effect of HgCl_2 _using the liquid cultures. We observed a significant difference in the growth profiles of wild‐type and *slr0701‐*mutant cultures, when incubated with 500 nM HgCl_2_. However, at 750 nM concentration both the strains exhibited similar growth phenotypes (Figure A3). This indicated that mercury‐induced expression of *slr0701 *gene seems to be crucial for *Synechocystis *cells to tolerate inorganic mercury. The functional complementation of *slr0701*‐mutant cells by the *slr0701 *gene explains that the slow growth phenotype observed in the presence of mercury was due to the inactivation of *slr0701*.

### 3.4 Effect of HgCl_2_ on photosynthetic activity due to mutation in *slr0701*

**Figure 3:**
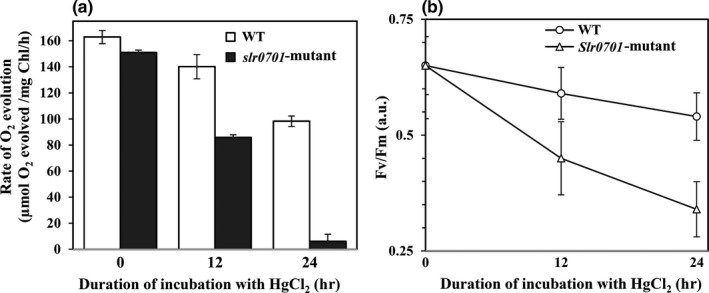
Changes in the photosystem II activity in *Synechocystis* wild‐type and *slr0701 *mutant cells during incubation with HgCl_2_. *Synechocystis* wild‐type and *slr0701 *mutant cells were grown to 0.6 at OD_730nm_ and then added 500 nM HgCl_2_. Collected cells before addition and 12 and 24 hr after addition of HgCl_2 _for measuring PSII activity. (a) Rate of oxygen evolution monitored by oxygraph in *Synechocystis* wild‐type and *slr0701 *mutant cells, (b) Changes in the *Fv/Fm *ratio in response to HgCl_2 _treatment. Mean values ± *SD* were calculated from three independent experiments

We measured the PSII activity in the wild‐type and *slr0701*‐mutant cells to analyze the extent of damage caused by mercury on photosynthetic performance due to inactivation of *slr0701 *gene. We used both oxygraph as well as relative *Chl a* fluorescence kinetics to analyze PSII activity. At optimal growth conditions, no significant difference in the PSII activity was observed (from water to PBQ) between wild‐type and *slr0701‐*mutant cells (Figure 3a). The PSII activity was decreased in both the WT and *slr0701*‐mutant cells when incubated with 500 nM HgCl_2 _for 12 and 24 hr, respectively. There was about 40% loss observed in the PSII activity of wild‐type cells at 24 hr of incubation with mercuric chloride, whereas the *slr0701*‐mutant cells showed 90% decrease in its PSII activity at the same point of incubation time (Figure 3a). Both wild‐type and *slr0701‐*mutant cells exhibited the characteristic OJIP fluorescence transient upon illumination of dark‐adapted cells. Minimal fluorescence (*Fo*) was observed to be almost constant with the increase in time of incubation with HgCl_2_, whereas variable fluorescence (*Fv*) and maximal fluorescence (*Fm*) was observed to be decreased significantly (data not shown). The *Fv/Fm *ratio, which reflects the activity of photosystem II was decreased in both the wild‐type and *slr0701‐*mutant cells when incubated with 500 nM HgCl_2_. About 17% decrease in *Fv/Fm *ratio was observed in wild‐type cells at 24 hr of incubation whereas the *slr0701*‐mutant cells showed about 48% decrease in *Fv/Fm* ratio at the same point of incubation time (Figure 3b). A decrease in the fluorescence yield due to mercury treatment can be attributed to inhibition of electron flow at the oxidising site of PS II (Lu & Vonshak, 2002). Such inhibition of electron flow, that is, reduced PSII activity due to incubation of cells with HgCl_2 _was much higher in the *slr0701‐*mutant than wild‐type cells. Data from both oxygraph and *Chl a *fluorescence kinetics are consistent and a rapid decline in PSII activity under HgCl_2_ stress in *slr0701‐*mutant clearly indicates severe damage in the photosynthetic machinery as compared to wild‐type cells.

### 3.5 Inactivation of *slr0701 *changes the mercury‐induced expression *mer *genes

**Figure 4:**
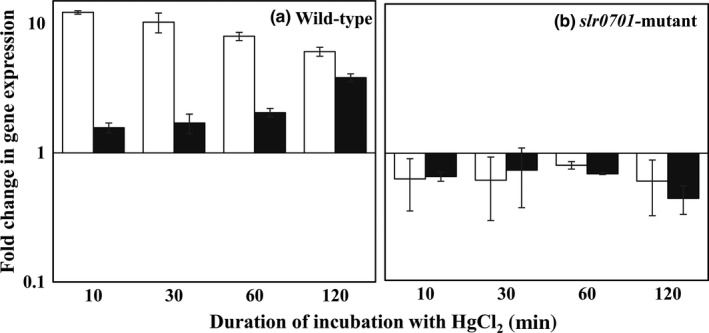
Change in the transcript levels of *slr0701 *and *slr1849 *during HgCl_2_ treatment. Fold change in gene expression for *slr0701 *(open bar) and *slr1849* (gray bar) were analyzed by qRT‐PCR during incubation with 500 nM HgCl_2_, (a) Expression changes in wild‐type, (b) Expression changes in *slr0701‐*mutant strain

The *slr0701 *and *slr1849 *mRNA levels were analyzed by real‐time PCR in the wild‐type and *slr0701*‐mutant cells. The expression of both *slr0701* and *slr1849 *genes was upregulated during incubation of the cells with HgCl_2_. Incubation of wild‐type cells with 500 nM HgCl_2_ resulted in upregulation of mRNA levels to 12.2 ± 0.27 of *slr0701 *and 1.5 ± 0.13 of *slr1849 *within 10 min. While the *slr0701 *mRNA levels were maintained to be high throughout the period of incubation, *slr1849 mRNA *levels were gradually increased to 3.81 ± 0.27 fold by 120 min (Figure 4a). In contrast, induced expression of neither *slr0701 *nor *slr1849 *was observed during the incubation of *slr0701‐*mutant cells with HgCl_2_. In fact, there was a little, but the significant downregulation of the expression of these genes was noted in the *slr0701‐*mutant (Figure 4b). The results indicate that upon inactivation of *slr0701* the expressions of its own gene and *slr1849 *gene were altered.

### 3.6 Slr0701 protein levels were enhanced due to the presence of HgCl_2_

**Figure 5:**
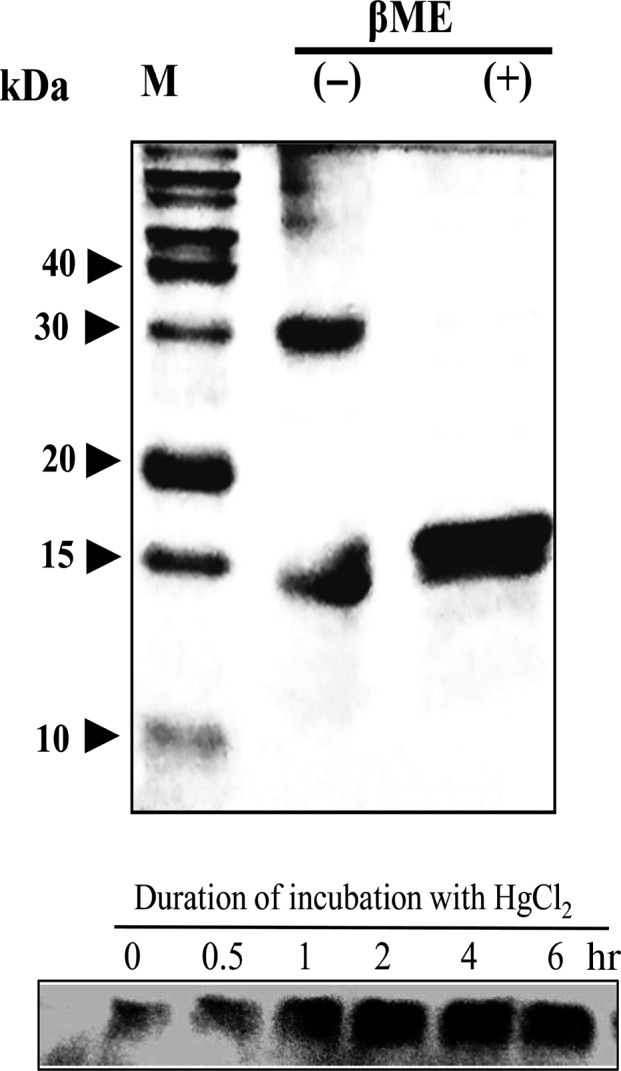
Homodimeric state of overexpressed and purified 6x‐His–Slr0701 protein. (a) SDS‐PAGE (15% gel) separation of purified 6x‐His–Slr0701 protein. *β*ME (−), in the absence of 2 mercaptoethanol; *β*ME (+), in the presence of 2 mercaptoethanol; M, protein markers 5–250 kDa. (b) Immunodetection of Slr0701 protein in the soluble fractions extracted from wild‐type and *slr0701‐*mutant *Synechocystis* cells upon 500 nM HgCl_2 _treatment for the indicated time; Slr0701, purified 6x‐His–Slr0701 was detected using an anti‐Slr0701 antibody

Slr0701 was induced and purified as a 6x‐His–Slr0701 protein from *E. coli* Rosetta™ (DE3). The 6x‐His–Slr0701 protein was resolved as a single protein band at ~17.3 kDa on SDS‐PAGE. Under semi‐denaturing condition (without 2‐mercaptoethanol), Slr0701 protein appeared near 30 kDa, indicating its probable existence as a dimer (Figure 5a). The Slr0701 protein was detected in soluble fraction, but not in the insoluble fraction of the lysate (Data not shown). The anti‐Slr0701 antibody recognized Slr0701 at a molecular mass nearly at 16 kDa in the wild‐type cells. Slr0701 protein was not recognized in *slr0701*‐mutant as anticipated. Figure 5b shows the immunodetection of Slr0701 in the wild‐type. There was a gradual increase in the Slr0701 protein levels during the course of incubation with HgCl_2_, which is in consistent with the upregulated gene expression of *slr0701* (increase in *slr0701 *mRNA levels) as was analyzed by qRT‐PCR.

### 3.7 Slr0701 binds to upstream of *slr0701* and *slr1849*

**Figure 6:**
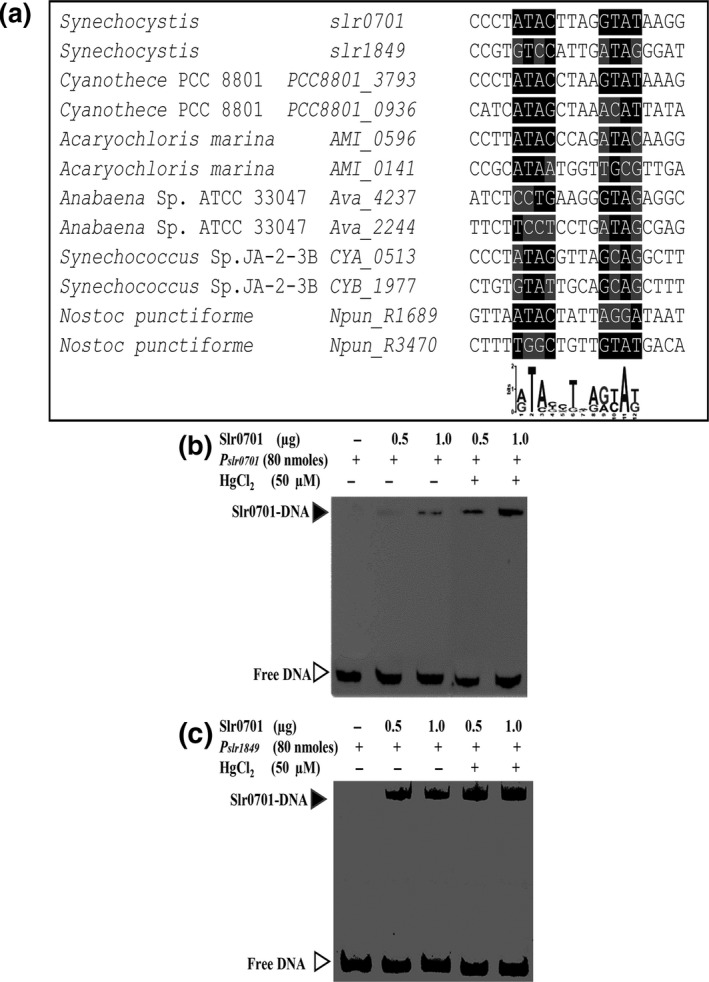
Prediction of a *cis‐*regulatory element in the upstreams of *mer *responsive genes and binding of Slr0701 to the upstream region of *slr0701 *and *slr1849. *(a) A common *cis‐*regulatory element was identified in the upstreams of *slr0701 *and *slr1849 *including their cyanobacterial homologs using *MEME suite *(http://meme.sdsc.edu/meme/cgi-bin/meme.cgi). Conserved inverted repeat region is highlighted in the alignment. Consensus representation of the inverted repeat is shown as a Logo below the alignment. Binding of Slr0701 protein to the upstream DNA fragment of *slr0701 *(b) and *slr1849 *(c) ORFs. A gel mobility shift assay was performed with 0.5 and 1 µg of 6x‐His‐Slr0701 and upstream DNA fragments covering the *cis‐*acting element were used in binding reaction. A 231 bp DNA upstream of *slr0701* starting from −1 to −231 bp with respect to the translation start site was used in (b). A 230 bp DNA upstream of *slr1849* starting from −1 to −230 bp with respect to the translation start site was used in (c)

As the expression of distantly located *slr0701 *and *slr1849 *genes were affected by the *slr0701* inactivation, a common cis‐regulatory binding element for Slr0701 protein is expected in the upstream of these genes. Using MEME motif discovery tool, we identified a common conserved *cis‐*regulatory element not only in the upstream of *slr1849 *and *slr0701 *genes, but also in the upstream of their homologs from other cyanobacterial species (Figure 6a).

**Figure A4:**
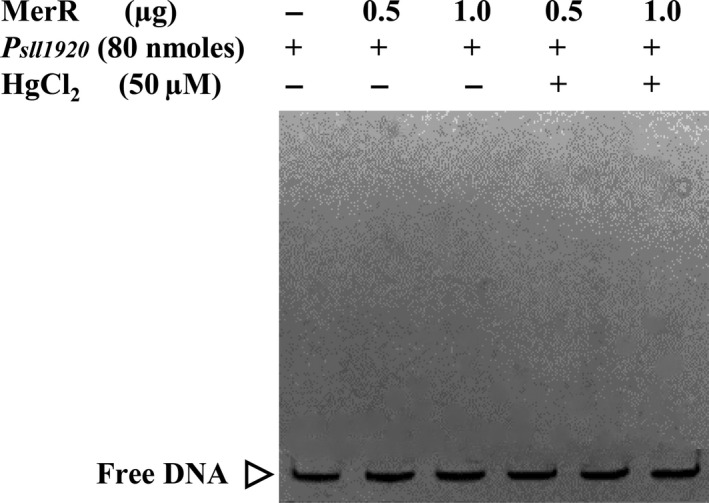
Binding of Slr0701 protein to the upstream DNA fragment of *sll1920* ORF. A gel mobility shift assay was performed with 0.5 and 1 µg of 6x‐His‐Slr0701 protein and 80 nmoles of upstream DNA fragment of *sll1920* in a binding reaction. A 201 bp DNA upstream of *sll1920 *starting from −1 to −201 bp with respect to the translation start site was used in the binding reaction

The binding of Slr0701 to the upstream of *slr0701 *and *slr1849 *with Slr0701 protein was studied using EMSA. Purified 6x‐His–Slr0701 protein retarded the electrophoretic mobility of the DNA fragments having P*_slr0701_* and P*_slr1849 _cis*‐acting elements, and intensities of retarded DNA bands were increased with the amount of protein used for incubation (Figure 6b,c). Importantly, in the presence of 50 µM of HgCl_2_, the intensity of retarded DNA‐protein complex was more compared to the intensity of the same in the absence of HgCl_2_. This result indicated that Hg^2+^ enhances the binding of Slr0701 to its target DNA binding site. In order to confirm the specificity of binding to *slr1849 *and *slr0701, *we used an unrelated DNA upstream (*sll1920) *in the presence and in the absence of HgCl_2 _(Figure A4). The 6x‐His‐Slr0701 did not bind to the unrelated‐DNA fragment confirming its specificity to the upstream of *slr1849 *and *slr0701.*

### 3.8 Binding kinetics of Slr0701 in the upstream of *slr0701*

**Figure 7:**
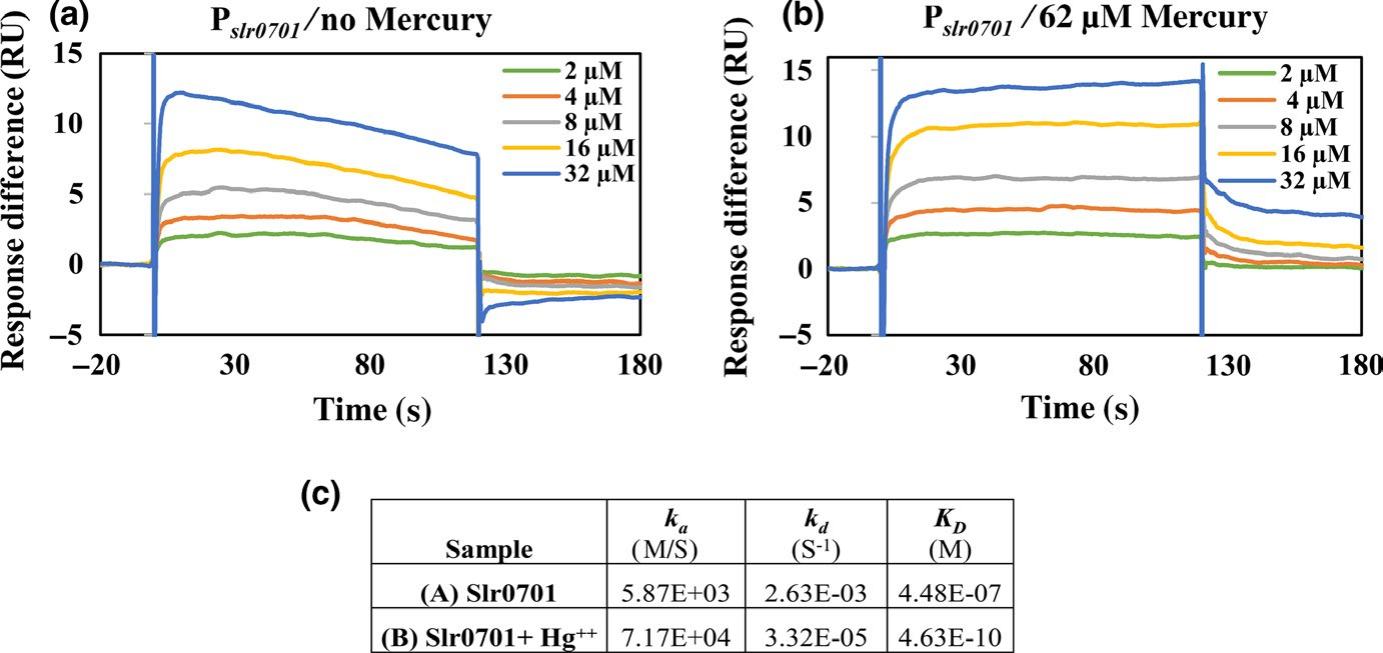
Sensorgrams obtained from surface plasmon resonance (SPR) analysis of binding of Slr0701 to the immobilized (a) P*_slr0701_* in the absence (b) P*_slr0701_* in the presence of 62 µM HgCl_2_. The concentrations of the analyte, Slr0701 used in each run is presented in the figure labels with the corresponding colors of the sensorgram. RU, relative units. (c) The association (k_a_), dissociation (k_d_) and equilibrium dissociation constants (K_D_) calculated form sensorgrams

We further analyzed the interaction between the upstream of *slr0701* and Slr0701 protein using SPR, as there was a significant increase in Slr0701 binding to P*_slr0701_* in the presence of HgCl_2_. We performed a binding analysis with varying concentrations of Slr0701 protein against the immobilized P*_slr0701_* DNA (Figure 7a). When the titration was repeated in the presence of 62 µM mercuric chloride, there was an increase in the response (RU). It is clear that association rate (*k_a_*) of the Slr0701 with immobilized DNA fragment was faster and the dissociation rate (*k_d_*) was observed to be slower in the presence of HgCl_2 _(Figure 7b)_. _The equilibrium dissociation constant (*K_D_*) evaluated from the kinetic traces showed that Slr0701 has nearly 1,000 times greater affinity to the promoter in the presence of HgCl_2 _(Figure 7c). This complements with the EMSA results where mercury enhanced the binding between the Slr0701 protein and upstream of *slr0701* (Figure 6b).

### 3.9 HgCl_2_ induces transcription of mercury responsive genes

**Figure 8:**
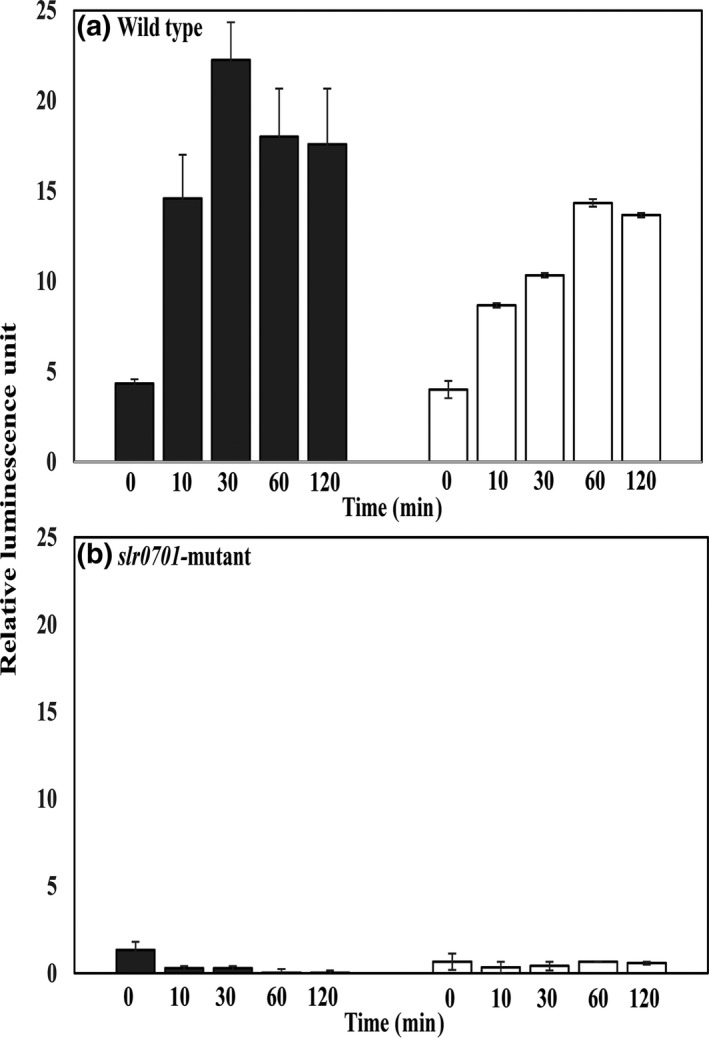
Mercury‐dependent changes in the activity of the *slr0701 *and *slr1849* promoters. *Synechocystis *wild‐type (a) and *slr0701‐*mutant cells (b) were grown in BG‐11 medium until the OD730_nm_ of the cultures reached to 0.4 and then treated with 500 nM HgCl_2_. Promoter activities were measured in terms of luminescence. P*_slr1849_*::LuxAB, *slr1849 *promoter‐reporter DNA construct (open bar)*; *P*_slr0701_*::LuxAB, *slr0701 *promoter‐reporter DNA construct (gray bar)

We monitored the activities of P*_slr0701_* and P*_slr1849 _*promoters in the wild‐type and *slr0701—*mutant cells in the presence and the absence of HgCl_2, _in order to examine the role of HgCl_2_ and Slr0701 in transcriptional activation of *merA*. The P*_slr0701_* and P*_slr1849 _*promoters were independently fused to a *luxAB *reporter gene and introduced into both *Synechocystis *wild‐type and *slr0701‐*mutant cells*. *These modified strains were used for monitoring activities of the promoters. A weak luminescence signal was detected in the wild‐type cells for P*_slr0701 _*and also for P*_slr1849_* promoters even in the absence of HgCl_2 _indicated their constitutive expression (Figure 8a). This result is consistent with previous reports where *merR *promoter found to be a weak promoter (Lund & Brown, 1989). The P*_slr0701_‐luxAB* in the wild‐type and in *slr0701*‐mutant showed 4.3 ± 2.4 and 1.3 ± 0.1 relative luminescence units (RLU) prior to the treatment with HgCl_2._ The P*_slr1849_‐luxAB* showed 4 ± 1.3 and 0.6 ± 0.3 RLUs prior to HgCl_2 _treatment indicating that the basal activity of P*_slr0701_* and P*_slr1849 _*promoters even in the absence of mercury. In the wild‐type cells, after the addition of HgCl_2 _there was an increase in luminescence signal suggesting that HgCl_2 _enhances the expression of *mer* genes in *Synechocystis *(Figure 8a)*. *Upon addition of HgCl_2 _to wild‐type cells, within 30 min the relative luminescence units were increased by four‐fold (22.2 ± 2.0) and three‐fold (10.3 ± 1.3) for P*_slr0701 _*and P*_slr1849_, *respectively indicating that both the promoters could get activated immediately in the presence of HgCl_2 _(Figure 8a). However, P*_slr1849_* activity was gradually increased as compared to the P*_slr0701_*, which is consistent with the qRT‐PCR results (see Figure 4a).

In contrast, the *slr0701*‐mutant cells harbouring plasmid DNA constructs having either P*_slr0701 _*or P*_slr1849_* promoter fused to *luxAB* genes did not show any luminescence signal either in the absence or in the presence of HgCl_2 _suggests that Slr0701 protein is necessary for mercury‐induced activation of its own gene expression as well as *slr1849* transcription (Figure 8b).

### 3.10 Circular dichroism analysis of structural changes in Slr0701

**Figure 9:**
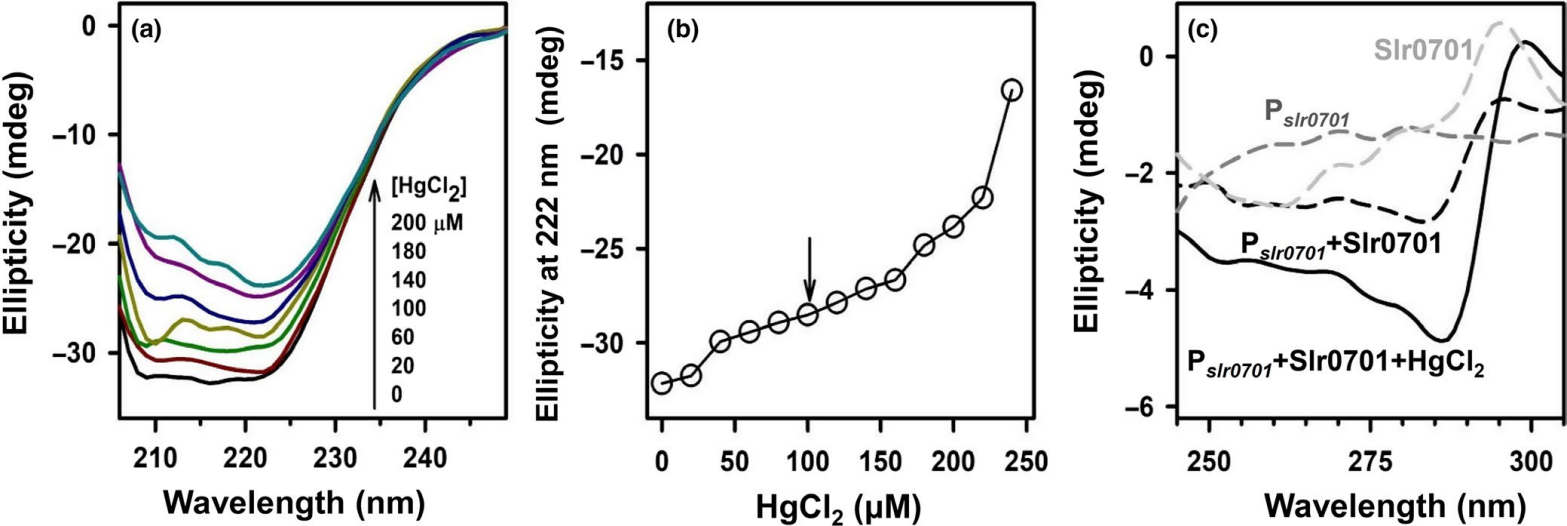
Circular dichroism analysis of Slr0701. (a) Far UV‐CD spectra of Slr0701 (20 µM) measured in varying concentrations of HgCl_2_. (b) The change in ellipticity of Slr0701 at 222 nm upon increasing the concentration of HgCl_2_ representing secondary structural loss in the protein. (c) Near UV‐CD spectra of Slr0701, P*_slr0701_*, P*_slr0701_*+Slr0701, and P*_slr0701_*+Slr0701+HgCl_2_

The structural changes in Slr0701 upon interaction with HgCl_2_ were analyzed using CD. In the far‐UV region (200–250 nm), a gradual decrease in negative ellipticity was observed, when the protein Slr0701 was titrated with increasing concentration of HgCl_2_. This suggests the relaxation in the secondary structure of the protein upon binding to HgCl_2 _(Figure 9a)_. _The ellipticity changes followed at 222 nm upon addition of HgCl_2_ is presented in Figure 9b. The initial changes saturating at 100 µM of HgCl_2 _might be attributed to the binding of HgCl_2 _whereas further sharp decline in the negative ellipticity could arise from denaturation of the protein.

In addition, the tertiary structural change in Slr0701 protein was analyzed in the near‐UV region (250–300 nm) upon binding with P*_slr0701_* with and without HgCl_2_. As compared to DNA—Protein alone, there was a significant increase in ellipticity in the presence of HgCl_2_ suggesting stronger binding of the protein with DNA in the presence of HgCl_2_ (Figure 9c).

## 4 DISCUSSION

Cyanobacterial species are widespread in both aquatic and terrestrial habitat and often expose to various toxic metals. Although the genome sequence information revealed the presence of a putative MerR (a transcriptional regulator) and MerA (mercury reductase) like genes in cyanobacteria, the organization of *mer *genes and regulation has not been studied. Interestingly, *merA *and *merR* genes are distantly located from each other in the cyanobacterial genomes, unlike in other bacterial phyla which contain five different *mer* genes forming an operon (Figure A2). In *Synechocystis, slr0701 *and *slr1849 *code for a putative transcriptional regulator (MerR) and a mercury reductase (MerA), respectively. The Slr0701 and Slr1849 proteins show greater similarities to homologs from *Geobacter *than well‐characterized homologs from *Bacillus *indicates that cyanobacterial *mer* genes might have horizontally transferred from these metal reducing species (Figure A1). Lack of several *mer *related genes and existence of only inorganic‐mercury reductase, MerA, a putative MerR transcription factor and their unique gene organization in all cyanobacterial species prompted us to elucidate the regulatory mechanism of mercury detoxification in *Synechocystis*.

**Figure 10:**
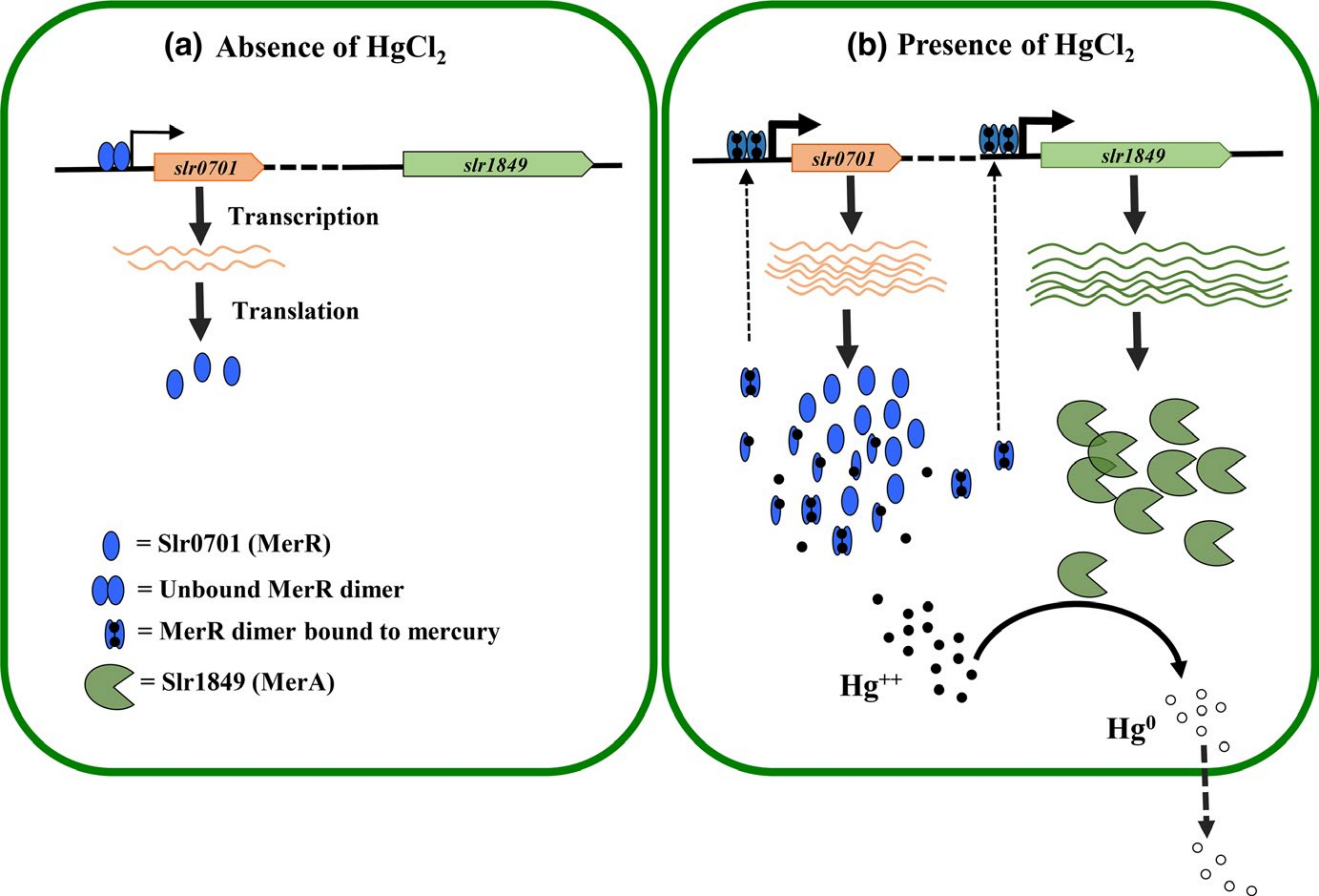
Schematic representation of mercury‐induced *merA *gene regulatory mechanism by a putative transcriptional regulator, Slr0701 in *Synechocystis* sp. PCC6803. (a) Slr0701 and Slr1849 genes are located far apart from each other in *Synechocystis *genome. Slr0701 is constitutively expressed and regulates its own gene expression. *Slr0701 *is transcribed from a weak promoter. (b) When cyanobacterial cells experience inorganic Hg^2+^, Slr0701‐Hg^2+^ complex binds to *cis‐*regulatory element located upstreams of its own ORF as well as *slr1849 *with greater affinity and leads to an induced expression. Thus Hg^2+^ bound Slr0701 activates the mercury‐dependent expression of Slr1849. Slr1849 being an inorganic‐mercury reductase converts Hg^2+^ to Hg^0^ and the volatile mercury is sent out of the cell

We inactivated the *slr0701 *that code for a putative MerR transcriptional regulator (Figure 1). Mercury is known to damage photosynthesis in cyanobacteria and higher plants (Bernier, Popovic, & Carpentier, 1993; Tangahu et al., 2011). We analyzed the extent of damage caused by HgCl_2_ on photosystem II activity. The *slr0701‐*mutant strain showed a severe reduction in photosynthetic activity and became relatively more sensitive to HgCl_2 _than wild‐type cells emphasizing its role in Hg^2+^ detoxification (Figures 2 and 3). Rapid and gradual upregulation of *slr0701 *and *slr1849 *mRNA levels were observed in the presence of HgCl_2 _(Figure 4). This induced gene expression was observed to be due to the binding of Slr0701 protein to the *cis‐*regulatory element in the upstream of its own ORF and *slr1849 *followed by transcriptional activation (Figures 5, 6 and 10)*. *Moreover, the *cis*‐regulatory element is found to be well conserved among most cyanobacterial *mer* homologs. In the presence of HgCl_2_, Slr0701 protein showed faster association and slower dissociation constants to the target DNA binding element (Figures 7 and 10). The *slr0701* and *slr1849 *are constitutively expressed. Upon addition of HgCl_2_, mercury‐bound Slr0701 enhances the transcription of these genes (Figures 8 and 10). In addition HgCl_2 _induced conformational changes in the protein and enhanced affinity to DNA leads to upregulation of gene expression (Figures 9 and 10). In the *slr0701‐*mutant, the mercury‐induced expression of mercury reductase, *merA *was not observed due to inactivation of *slr0701 *gene. Hence, the *slr0701*‐mutant became more sensitive to Hg^2+^ than wild‐type. Cyanobacteria contain only a well‐conserved transcriptional regulator, MerR and a mercury reductase, MerA. They are found to be distantly located in the genome which is a unique feature when compared to other bacterial phyla. However, MerR could still regulate the mercury‐dependent expression of MerA. The mechanism of gene regulation involving MerR is well‐conserved among all bacterial phyla.

## CONFLICT OF INTERESTS

None declared.

## AUTHORS CONTRIBUTION

D.K.S performed majority of the experiments and wrote the paper. L.B generated the mutant strain. K.T.N and D.K.S performed CD spectral experiments together. N.P.P analyzed the CD and SPR results and edited the paper. J.S.S.P conceived the idea for the project and wrote the paper with D.K.S.

## ETHICS STATEMENT

None required.

## Data Availability

The data that support the findings of this study are available from the corresponding author upon reasonable request.
